# The genetic proteome: Using genetics to inform the proteome of mycobacterial pathogens

**DOI:** 10.1371/journal.ppat.1009124

**Published:** 2021-01-07

**Authors:** Kathleen R. Nicholson, C. Bruce Mousseau, Matthew M. Champion, Patricia A. Champion

**Affiliations:** 1 Department of Biological Sciences, University of Notre Dame, Notre Dame, Indiana, United States of America; 2 Department of Chemistry and Biochemistry, University of Notre Dame, Notre Dame, Indiana, United States of America; 3 Boler-Parseghian Center for Rare and Neglected Diseases, University of Notre Dame, Notre Dame Indiana, United States of America; Carnegie Mellon University, UNITED STATES

## Abstract

Mycobacterial pathogens pose a sustained threat to human health. There is a critical need for new diagnostics, therapeutics, and vaccines targeting both tuberculous and nontuberculous mycobacterial species. Understanding the basic mechanisms used by diverse mycobacterial species to cause disease will facilitate efforts to design new approaches toward detection, treatment, and prevention of mycobacterial disease. Molecular, genetic, and biochemical approaches have been widely employed to define fundamental aspects of mycobacterial physiology and virulence. The recent expansion of genetic tools in mycobacteria has further increased the accessibility of forward genetic approaches. Proteomics has also emerged as a powerful approach to further our understanding of diverse mycobacterial species. Detection of large numbers of proteins and their modifications from complex mixtures of mycobacterial proteins is now routine, with efforts of quantification of these datasets becoming more robust. In this review, we discuss the “genetic proteome,” how the power of genetics, molecular biology, and biochemistry informs and amplifies the quality of subsequent analytical approaches and maximizes the potential of hypothesis-driven mycobacterial research. Published proteomics datasets can be used for hypothesis generation and effective post hoc supplementation to experimental data. Overall, we highlight how the integration of proteomics, genetic, molecular, and biochemical approaches can be employed successfully to define fundamental aspects of mycobacterial pathobiology.

## Introduction

Mycobacterial species have coevolved with humans over thousands of years [1]. Of the 188 distinct mycobacterial species, many are clinically relevant or emerging pathogens [2]. *Mycobacterium tuberculosis* is an obligate human pathogen that causes tuberculosis [3]. Several environmental mycobacterial species cause disease in humans and animals. While *M*. *tuberculosis* alone caused 1.5 million deaths in 2018 [4], infections involving other nontuberculous environmental species of pathogenic mycobacteria (nontuberculous mycobacteria (NTM)) are increasing, and in some countries including the United States, NTM infections outnumber those by *M*. *tuberculosis* [5,6]. These largely opportunistic pathogens, including *M*. *abscessus*, *M*. *kansasii*, and the *M*. *avium* complex (MAC), cause lung disease and other systemic infections sometimes associated with medical implants [6,7].

The bacillus Calmette–Guérin (BCG) vaccine is the only vaccine against *M*. *tuberculosis* infection, and its effectiveness in adults is poor [8]. There are no vaccines against NTMs, and diagnostic and treatment options are limited. *M*. *tuberculosis* has become increasingly resistant to the antibiotic cocktails used to treat tuberculosis [4]. NTM pathogens harbor natural antibiotic resistance to many of the drugs used to treat tuberculosis [9,10]. *M*. *abscessus* isolates, for example, are intrinsically resistant to tetracycline at concentrations 500-fold higher than observed for *M*. *tuberculosis* [11]. There remains a critical need for new diagnostic, therapeutic, and vaccine targets, which benefits from understanding the basic physiology and virulence mechanisms in mycobacteria.

The proteome is the collection of proteins associated with a cell, tissue, or organism under a set of conditions. Recent advances in mass spectrometry (MS) and data-processing have enabled an expansion from static snapshots of the proteome to one that is more temporal, quantitative, spatial, and targeted. Technical and methodological advancements in mass spectrometers, protein fractionation/separations, preparation/enrichment and informatics have made proteomics a fast and reliable discipline for whole proteome studies. The strength of these approaches lies in the ability to generate large datasets biased predominately by protein abundance and targeted enrichment. Hypothesis-driven biological research provides the context to these deep and global approaches.

Proteomic datasets can be powerful tools for generating new hypotheses or testing existing ones. Quantitative proteomics is an encompassing term for several isotopic and non-isotopolog–based strategies to quantify liquid chromatography–mass spectrometry (LC-MS) data [12–15], which are routinely used to address changes in protein levels in pathogenic mycobacterial studies. Quantifying LC-MS data is typically performed by comparing the intensity or peak area for proteotypic peptides across conditions and replicates. These approaches have been widely applied to microbial studies and mycobacteria [16–18]. The proteomic analysis of hypo- and hypervirulent strains of *M*. *tuberculosis* identified differential protein expression after mouse infection providing new insight into mycobacterial virulence factors [19]. Numerous other analyses of the secreted proteins of *M*. *tuberculosis* identified protein virulence factors present at higher levels in hypervirulent strains compared with *M*. *tuberculosis* laboratory strains [20–22]. These “census-style” experiments report the presence and/or abundance of proteins for comparative and declarative values. Although there are several ways to develop context for proteomic experiments, we think using comparative proteomics in infection models and combining the strength of genetics and genetic content to augment the proteome are the most productive approaches.

**Fig 1:**
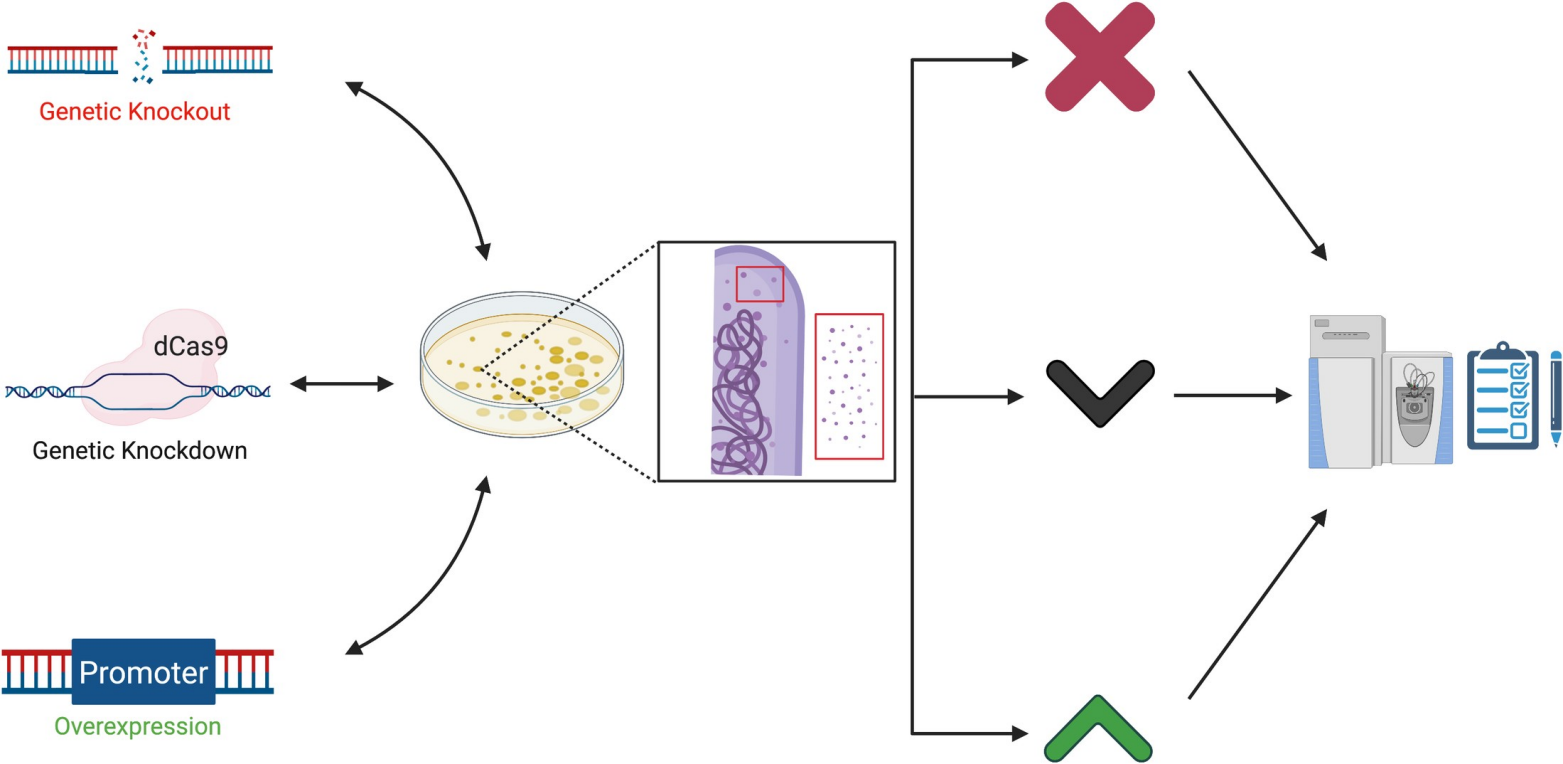
The genetic proteome. Manipulating the genome using genetic approaches (left) alters the cell-associated and naturally enriched proteomes derived from bacteria resulting in protein loss (X) or increased or decreased protein levels (up and down arrows). These are measured and confirmed or rejected using proteomic approaches (right). Together classical and analytical approaches further our understanding of how specific genes affect mycobacterial physiology and virulence. (Created with BioRender). dCas9, dead Cas9 protein.

Physiological context can be obtained through the use of classical bacteriological approaches, including molecular genetics and biochemistry, and more recently “-omics” approaches. Our concept of the “genetic proteome” is underscored by the deliberate use of genetics, or other classical approaches, to alter the measurable proteome (Fig 1). The genetic proteome differs from multi-omics and proteo-genetics approaches. Multi-omics combines multiple “omes” to provide depth, while proteo-genetics typically relies on existing natural genetic diversity and brute-force computation to define the search space for MS-based proteomics [23–25]. We are suggesting that incorporating classical and proteomic approaches improves our understanding of organismal physiology, more so than either approach independently [26]. By leveraging the awesome power of genetics, mycobacterial researchers using proteomics, as other microbial proteomics fields, enjoy near-perfect controls for their analyses. Exploiting both mycobacterial genetics and proteomics has allowed researchers to link changes in single or global protein levels or modifications to specific mycobacterial genes and pathways. In this review, we consider several types of proteomic analyses in mycobacteria, as well as the contributions these approaches have made.

## Exploiting the awesome power of mycobacterial genetics

Researchers using *M*. *tuberculosis* and some NTMs benefit from robust genetic approaches for dissecting the role of specific genes and pathways in disease. Despite differences in environmental niche, host range, and disease etiology, fundamental mechanisms of virulence are well conserved between *M*. *tuberculosis* and NTMs [27–30]. Both *M*. *tuberculosis* and NTMs also share physiological pathways with the nonpathogenic mycobacterial species *M*. *smegmatis* which is similarly genetically tractable, and also exhibit conjugation [31,32]. Therefore, studies in nonpathogenic and nontubercular pathogenic mycobacterial species as well as those in *M*. *tuberculosis* contribute to defining fundamental aspects of mycobacterial biology [9]. Many mycobacterial species are robustly and efficiently transformed with DNA, with several available markers for antibiotic resistance. Genetic knockouts and knockdowns, as well as gene overexpression, are now routine manipulations in several (but not all) mycobacterial species. Numerous knockout approaches include suicide plasmid [33–37] and phage-mediated allelic exchange [38], CRISPR interference (CRISPRi) [39–41] oligonucleotide-mediated recombineering followed by Bxb1 integrase targeting (ORBIT) [42], and recombineering approaches [43]. There are also several robust transposon platforms available that have been widely exploited to understand mycobacteriology both in vitro and in host models for infection [44–46]. Foundational examples of using this approach include the transposon site hybridization (TraSH) and signature tagged mutagenesis (STM) transposon platforms. An example of TraSH in *M*. *tuberculosis* identified essential and conditionally essential genes, and those required during host infection [44,47,48]. An example of STM in *M*. *tuberculosis* revealed roles for phthiocerol dimycocerosate (PDIM), a cell wall–associated lipid [46], and the ESAT-6 system -1 (ESX-1) secretion system in mycobacterial virulence [49]. The suicide plasmid or phage-based allelic exchange approaches and ORBIT ideally result in unmarked deletions that impact the expression of a single mycobacterial gene [42]. Numerous studies reporting targeted unmarked deletions of genes in pathogenic and nonpathogenic mycobacterial species have identified roles of individual genes in various physiological and virulence processes (some exemplar publications include [50–55]). CRISPRi approaches rely on targeting the dead Cas protein (dCas) complex to prevent or reduce gene expression and can be polar on downstream genes [39]. CRISPRi silencing can be applied to conditionally silence individual or multiple genes or be used for generating high-density silenced libraries [39,56]. Importantly, the dCas system is inducible and can be used to study essential mycobacterial genes, for example, to define their role in physiology or as potential therapeutic targets [57–59].

To demonstrate that the genetic knockout caused the observed phenotypes, there are several options for genetic complementation by restoration of gene expression, including plasmids that integrate at 2 different phage attachment sites and episomal plasmids with both low-level and high-level constitutive promoters [60–62]. There are also several inducible promoter systems and riboswitch platforms that can be used for genetic complementation or to perform depletion studies to study essential mycobacterial genes [63–71]. Essential mycobacterial genes are often studied as potential drug targets. For example, the genetic depletion of the *M*. *tuberculosis* Rho transcription termination factor using anhydrotetracycline inducible repression led to widespread transcriptional dysregulation and bacterial growth defects, demonstrating that Rho is essential and a potential target of novel antitubercular drugs [72]. Therefore, there are several forward genetic approaches that are accessible to the mycobacterial community and facilitate understanding how specific genes contribute to basic mycobacterial physiology and virulence. These genetic approaches have been applied to alter the measured proteome (Fig 1).

## Targeted and comprehensive proteomic approaches define the mycobacterial proteome and its modifications

**Fig 2:**
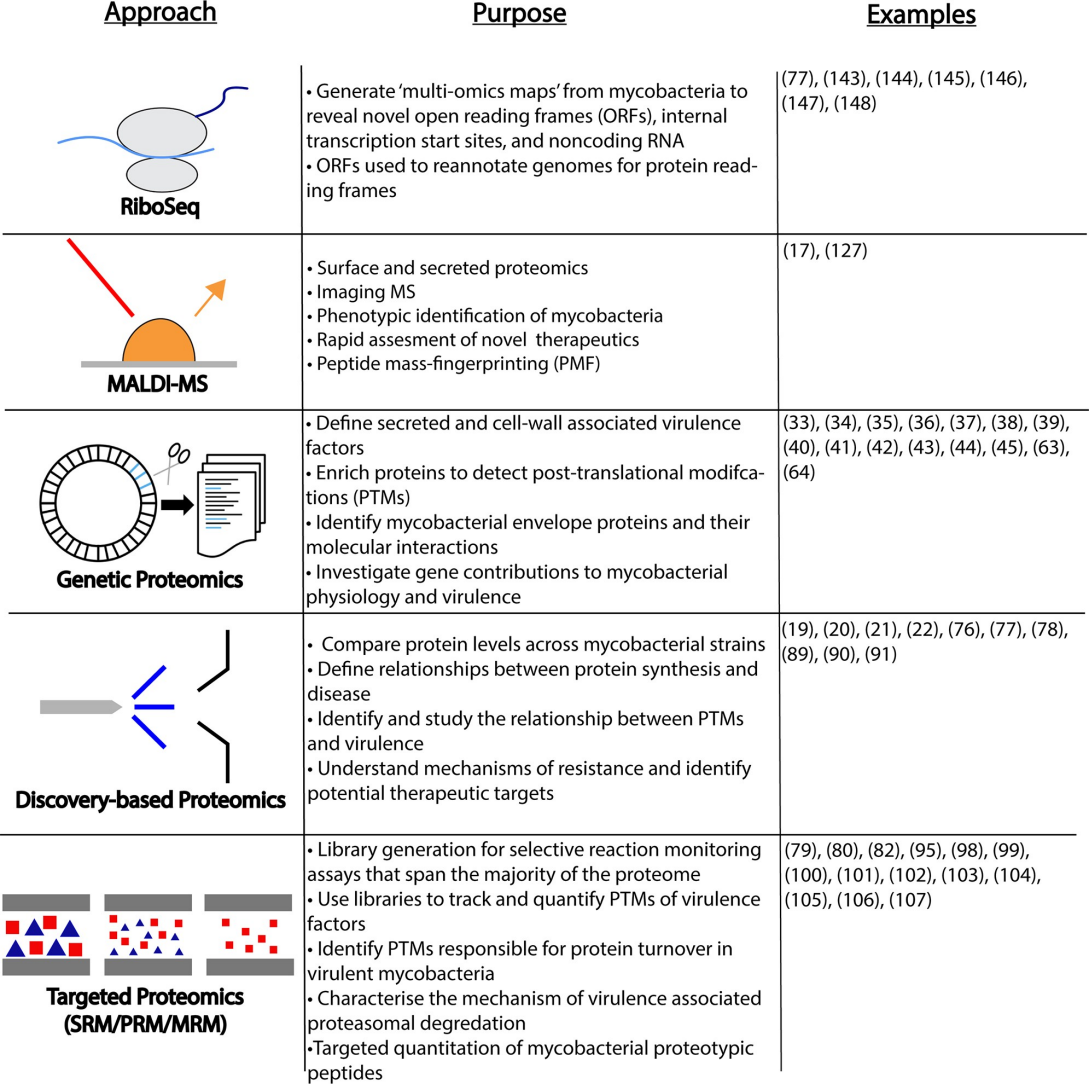
Proteome-centric approaches used in the study of mycobacteria described in this review. Listed are 5 major approaches, their associated measurements, and representative references. Ribo-seq methods measure the “translatome” and have been applied to annotate and define the mycobacterial genome landscape. MALDI-MS–based approaches are more rapid and are used in screening approaches. Genetic proteomics uses alterations in the genome to manipulate the measured proteome. Discovery and targeted proteomics approaches measure and quantify proteins and their modification from complex and enriched cellular material. Examples list numbers of relevant references. MALDI-MS, matrix-assisted laser desorption/ionization mass spectrometry; MRM, multiple reaction monitoring; ORFs, open reading frames; PMF, peptide mass-fingerprinting; PRM, parallel reaction monitoring; PTMs, posttranslational modifications; Ribo-seq, ribosome sequencing; SRM, selected reaction monitoring.

MS-based proteomics is a diverse set of approaches applied to study individual proteins, groups of proteins, or the entire proteome of the bacterial cell (Fig 2). So-called “bottom-up” proteomics, the most applied method for proteomics studies, creates peptides from proteins typically using a protease like trypsin [73]. The mass-to-charge ratio of the peptides are measured (MS1) before further fragmentation in a tandem manner to generate MS-MS daughter ions. Peptides fragment by predictable mechanisms, and the masses of the fragment ions are used to infer the protein from which it derived. This process is performed most commonly by spectral mass-matching, and occasionally by de novo sequencing [73]. The bottom-up approach has been used as a building block for both discovery and targeted proteomics and widely applied to basic mycobacterial research [52,74,75]. These discovery-based methods have aided in profiling clinical isolates of *M*. *tuberculosis* and distinguishing the relationship between protein synthesis and active disease [19,76–78].

Targeted proteomics is an approach where identification/detection of specific proteins/peptides is known prior to LC-MS/MS acquisition. It provides substantial gains in sensitivity and specificity (signal-to-noise ratio) at the expense of target density [79–82]. Because quantitative data obtained from targeted proteomics is more accurate and precise, it is the preferred approach when determining absolute quantitative abundance using stable heavy-isotope dilution Absolute Quantification (AQUA) style approaches [83,84]. Stable heavy isotope dilution relies on MS acquisition referred to as selected/multiple reaction monitoring (SRM/MRM), or parallel reaction monitoring (PRM). Although numerous MS instruments are capable of performing targeted proteomics, triple quadrupoles are the most common, sensitive, and precise platform for performing these experiments, and they are extensively applied to study mycobacterial pathogenesis [18,79,80,82]. Targeted proteomics is the dominant form of LC-MS/MS in clinical proteomics used for diagnostic, drug, and detection [85–87].

Both targeted and non-targeted proteomics methods have identified protein posttranslational modifications (PTM) in mycobacteria. Mycobacteria use PTMs to regulate protein function and activity. Glycosylation, phosphorylation, lipidation, formylation, pupylation, acetylation, and methylation have been identified in *M*. *tuberculosis* [88,89]. Several studies have elucidated extensive networks of reversible (S/T/Y) protein phosphorylation in mycobacteria [90–93], during mycobacterial infection [91,94], and the identification of irreversible protein N-terminal acetylation as an abundant PTM in mycobacteria associated with virulence and survival [17,95].

Targeted approaches have made it possible to create a global *M*. *tuberculosis* library which contains SRM assays covering 97% of all annotated proteins for *M*. *tuberculosis* and 72% of the entire proteome [82,96]. Libraries such as these allow other researchers access to deeper mechanistic insight into *M*. *tuberculosis* treatments that directly benefit tuberculosis patients. In previous work, libraries of MRM/SRM transitions to quantify mycobacterial protein levels have allowed targeted approaches to identify and measure posttranslational modifications of virulence factors that could be clinically relevant signatures of infection [79,80].

In addition to modification, protein turnover by proteasomal degradation is essential for *M*. *tuberculosis* virulence in mice [97] and during stress under nitrogen starvation and DNA damage in *M*. *smegmatis* [98–100]. Protein turnover is regulated by both protein structure and protein modifications [101–103]. Affinity purification, 2D gel electrophoresis (2DGE), and targeted proteomics in *M*. *tuberculosis* led to the identification of pupylation, which is the addition of the prokaryotic ubiquitin-like protein (Pup) to protein lysine residues which targets proteins for proteasomal degradation [104–107]. Emerging proteomics techniques continue to increase throughput in the detection of a variety of PTM events.

## The genetic proteome defines secreted and cell wall–associated virulence factors

Proteomes can be fractionated to study proteins localized to specific compartments within the cell or those secreted by the bacteria to the cell surface or into the extracellular environment. Fundamentally, secreted proteomes or “secretomes” are a “natural fraction” of the proteome (Fig 2). Secretomes are inherently less complex than the whole cell proteome and often enriched for proteins important for physiology and virulence. As such, secretomes have been widely exploited to generate dense biological context in particular for mycobacteria. One of the largest challenges in the area of secreted proteomics in mycobacteria is the tendency for a subset of the bacteria to lyse during routine culturing. Lysis contaminates the secreted proteome with cytosolic contents. This has been addressed by the careful control of growth when proteomics is intended and subtractive controls for cytosolic contaminants [16,108–110].

Proteomic studies on culture filtrates (proteins secreted into the culture media during mycobacterial growth in vitro), when combined with powerful and precise genetic approaches, were primarily responsible for the elucidation of the several of known ESX (type VII)-secreted proteins, which are essential for mycobacterial pathogenesis [79,80,111–114]. A powerful example of the applied genetic–proteome approach has been altering the secreted proteome with genetics to identify the secreted substrates associated with specific secretory systems. For example, defining the secretome in the presence and absence of the *esx-1* genes encoding the ESX-1 secretory system, numerous conserved ESX-1-dependent substrates were identified including EspA, EspF, EspJ, EspK, and PPE68 [79,80,111], as well as other substrates unique to the nontubercular species [112,114,115]. Similar strategies have been widely applied to identify and study the substrates of other cell-associated and alternative secretion systems in mycobacteria, including but not limited to the SecA2 system [116–118]. It is important to note that many bacterial proteins are not amenable to detection using antibodies. Moreover, epitope tagging and fusion to ESX-1 substrates prevents secretion through the ESX-1 system or diminishes function to an unacceptable degree. Therefore, proteomics approaches are in some cases the only way to identify these important virulence factors.

Another key finding regarding mycobacterial protein secretion was that protein secretion is not “on or off.” The advent of label-free quantitative (LFQ) proteomics approaches allowed for the increased detection of low abundance peptides in secreted fractions, revealing the potential for intermediate secretion phenotypes in transposon mutant strains [80,111].

One limitation to using proteomics approaches when studying the secreted proteins of mycobacterial pathogens is the difficulty in detecting mycobacterial proteins in the host. Instead, there have been several clever approaches to contextualize existing proteomics data and verify that proteins secreted by mycobacteria in vitro are also secreted in the host [119–121]. These approaches could also reveal mycobacterial proteins only secreted within the host and not when mycobacteria are grown in vitro.

In addition to the secretome, proteins associated with the cell envelope and the cell surface, as well as those in secreted microvesicles, are essential for mycobacterial physiology and pathogenesis [122]. Genetic–proteome approaches have yielded important information into the protein content of mycobacterial membranes and how these proteins interact with each other [123–126]. Whole colony proteomics approaches contributed to measuring virulence factors associated with the mycobacterial cell surface, in the presence and absence of mutations or deletions in genes encoding secretory systems [127]. Defining the cell wall proteome has involved biochemical approaches including cell surface enzymatic [117,128,129] or hydrolysis shaving [130] followed by MS to identify surface-exposed mycobacterial proteins. However, cell surface techniques can be ineffective. Biochemical cell wall fractionation is not only often more robust, but can also be more challenging than modifying the cell surface. Coupling cellular fractionation of both wild-type and SecA2-deficient *M*. *tuberculosis* strains with LFQ MS revealed that the mycobacterial cell wall proteins and solute transporters require the SecA2 system for localization [118]. One promising emerging approach is ascorbate peroxidase (APEX) proximity-based biotinylation to selectively label and enrich proteins within the cell wall. This approach has been coupled with proteomics approaches to identify protein–protein interactions and inform molecular pathways to promote cell wall biogenesis [131–133]. Likewise, chemical probes for detecting direct interactions between proteins in the mycobacterial cell envelope have been coupled to quantitative proteomics. These approaches have revealed more than 100 envelope proteins and their binding partners in *M*. *smegmatis* [134]. Another interesting example of the genetic proteome is a recent study in which genetics were used to alter the proteome of microvesicles produced by *M*. *tuberculosis*. By using genetic deletions that alter *M*. *tuberculosis* signal transduction followed by microvesicle isolation and proteomics, the authors defined specific proteins regulated by the Pst1/SenX/RegX signal transduction system that are targeted to microvesicles [135].

## The genetic proteome and antibiotic susceptibility and resistance

In addition to defining virulence mechanisms, using genetics to alter the proteome can also provide insight into antibiotic sensitivity and resistance. For example, protein overexpression can alter the proteome and impact mycobacterial drug sensitivity and resistance. A recent example of this type of study was performed in *M*. *bovis*. A latency-related universal stress protein (USP, BCG_2013) was overexpressed in *M*. *bovis*, which increased the efficacy of isoniazid (INH) [136]. Subsequent quantitative proteomics analysis revealed that USP overexpression resulted in 50 up-regulated proteins, including catalase peroxidase KatG, which is required for INH activation in the mycobacterial cell [136]. Similar overexpression coupled with proteomics approaches have been used to study lipid metabolism and vancomycin resistance in *M*. *smegmatis* [137].

Interestingly, clinical mycobacterial strains carry frameshift and nonsense mutations in the essential *rpoB* gene (for example, [137–139]). Strains with mutations in essential genes should be nonviable. However, frameshift and nonsense mutations in the *rpoB* gene, which encodes the β-subunit of RNA polymerase, cause resistance to rifampicin, because rifampicin kills bacteria by interacting with RpoB and blocking transcription [140,141].

By coupling genetic and proteomic approaches, it was recently shown that frameshifting of the *rpoB* gene is a mechanism used by pathogenic mycobacteria to generate resistance to antibiotics [142]. Importantly, LC/MS/MS was applied to demonstrate low levels of frameshift suppression of *rpoB* mutations, indicating that sometimes, what is encoded in the genome does not necessarily predict the resulting proteome [142]. The decoupling of the proteome from the genome underscores the necessity of combining approaches that alter the genome and measure the proteome.

## The genetic proteome and multi-omics reveal new insight into transcription and translation in mycobacteria

Combining classical and -omics approaches have shed new light on the genetic structure and the fundamental mechanisms of transcription and translation in mycobacteria. There are also several recent examples of integrative multi-omics methods combining transcriptomic, proteomic, and metabolomic approaches with genetics to interrogate mycobacterial physiology.

A recent study using differential RNA-seq, ribosome profiling, and proteomics of *M*. *abscessus* under different clinically relevant conditions generated “multi-omics maps,” revealing regulation by novel short open reading frames (ORFs), internal transcription start sites, and noncoding RNAs [143]. These short ORFs have been identified in *M*. *tuberculosis* and NTMs for individual genes and used to reannotate existing genomes for protein reading frames [77,144–148]. The majority of translation events occur on mRNA transcripts with a 5′ untranslated region (UTR) which includes a Shine–Delgarno ribosome binding site. mRNA transcripts lacking a 5′UTR and a ribosome binding site are referred to as “leaderless.” However, recent studies in *M*. *smegmatis* have investigated protein translation initiation through transcription start site mapping and selective detection of protein N-termini by MS, ultimately illustrating widespread leaderless translation in mycobacteria [144,145,147].

Transcription factors and mechanisms of RNA degradation also remain topics of intense investigation by mycobacterial researchers. Commonly used approaches include the genetic perturbation of a specific transcription factor gene followed by studying the resulting changes in gene expression (exemplar publications include [52,149,150]). Alternatively, transcriptional regulons have been defined using whole genome approaches including transcription factor overexpression followed by chromatin immunoprecipitation (ChIP) (exemplar publications include [151,152]). However, advances in biochemical, molecular, and proteomic approaches has improved the inverse of this process—the selective identification of proteins responsible for modulating transcriptional regulation through the direct binding to DNA or RNA. In this approach, rather than genetics, biochemistry is coupled to proteomics. Recent work in *M*. *smegmatis* used an affinity purification–mass spectrometry (AP-MS) approach to globally identify transcription-associated proteins. Here, chemical crosslinking and affinity purification of the RNA polymerase β-subunit led to the global identification of 275 transcription-associated proteins [153]. Of these proteins, 20 were not previously associated with nucleic acids [153]. The function of these transcription associated proteins could then be confirmed by coupling genetics and transcriptomics approaches as indicated above. This work exemplifies the utility of proteomics and molecular approaches in providing foundational regulatory classifications for mycobacterial proteins globally. In a similar approach, DNA affinity chromatography and proteomics approaches were used to enrich and identify proteins specifically bound to a promoter, resulting in the identification of a novel transcription factor required for the regulation of genes by the ESX-1 secretion system [150]. In this study, multi-omics techniques with traditional reverse genetics methodologies uncovered multitiered regulatory phenomena controlling a key protein transport system in mycobacteria.

Protein–protein interactions are commonly critical for the regulation of physiological processes and pathogenesis within the host. Targeted immunoprecipitation of single proteins has been used to assemble regulatory pathways across mycobacterial physiology and aid in protein characterization; these studies span many aspects of mycobacterial physiology, including virulence, metabolism, and persistence [154,155]. Immunoprecipitation and MS have long been used to identify novel binding partners of essential ATPases, illustrating the utility of affinity enrichment to discover protein–protein interactions necessary for cell survival and pathogenesis [79,156].

Finally, as part of a multi-omics approach, proteomics has been used to complement transcriptomics to define the molecular composition of the machinery that degrades RNA in *M*. *tuberculosis*, known as the “degradosome.” In this study, RNA–protein complexes were enriched, and components of the degradosome were identified by proteomics. Targeted genetic approaches were applied together with transcriptomics to confirm the role of putative degradosome complex components in global RNA turnover [157].

## What’s next in applying the genetic proteome to mycobacterial research?

As discussed above, harnessing the power of proteomics to its full extent requires effective integration with other approaches, database curation, and network correlation. Proteomic datasets generated from published studies are useful data mining resources that can be directly leveraged to inform future research. These datasets provide a wealth of information that can be used to generate new hypotheses that can be tested using classical approaches or supplement existing experimental data. Examples in recent literature illustrated the utility of tandem MS dataset mining in validating genomic mutations leading to chimeric protein production in *M*. *tuberculosis* clinical isolates [56,158]. However, variation in proteomics methodology, sample preparation, and physiological context can lead to ineffective data utilization. Database curation is 1 way to circumvent these issues. Data repositories and libraries, like the MRMaid database and SRMAtlas, have been constructed to store experimental data from SRM and MRM assays [159,160]. An *M*. *tuberculosis* proteome library has been stored on SRMAtlas encompassing SRM assays for 97% of all annotated *M*. *tuberculosis* proteins [82]. Table 1 describes representative datasets and repositories of SRM/MRM (targeted), bottom-up (non-targeted), and mRNA/ribosome sequencing (Ribo-seq (profiling))–based approaches from pathogenic mycobacteria utilizing proteo-genetic methodologies.

**Table 1:** Mycobacterial genetic proteome databases and datasets.

Name/Type	Location	Reference
Targeted Databases		
Peptide Atlas (*M*.*tb*)	SRM Atlas http://www.srmatlas.org/	Schubert et al., 2013 (82)
SRM Atlas (*M*. *tb*)	Peptide Atlas http://www.peptideatlas.org/	Schubert et al., 2015 (18)
MRM/SRM profiling of ESX-1 mutants in culture filtrate and cytosol	https://pubs.acs.org/doi/10.1021/pr500484w	Champion et al., 2014 (80)
*These are comprehensive MRM/SWATH datasets for targeted detection of M*. *tb in cells*, *host and filtrates*		
Non-targeted Datasets		
High-resolution proteogenomic analysis of *M*. *tb* H37Rv	Peptide Atlas PAe001767	Kelkar et al., 2011 (23)
	Proteome Xchange PXD010956	
Lineage-specific proteomes: virulence etc. large comprehensive MS/MS dataset	Proteome Xchange PXD020383	Yimer et al., 2020 (56)
*These are representative datasets from large-scale MS-MS/MS bottom-up profiling*		
mRNA / Ribosomal Profiling		
Ribo-seq of *M*. *smeg* and *M*. *Tb*	EMBL-EBI E-MTAB-2929	Shell et al., 2015 (144)
RNA-seq, RNA-seq of 5'PPP, proteomics	EMBL-EBI E-MTAB-1616	Cortes et al., 2013 (147)
	Proteome Xchange PXD000483	
*These studies identified the extensive leaderless and small ORF's population within Mycobacteria*		

Representative datasets and databases from *M*. *tuberculosis* (*M*. *tb*) studies, organized by proteome approach. A brief description, the type, reference, and current raw file location is included.

MRM, multiple reaction monitoring; MS, mass spectrometry; ORFs, open reading frames; Ribo-seq, ribosome sequencing; RNA-seq, RNA sequencing; SRM, selected reaction monitoring.

## Conclusions

Proteomics has considerably advanced the field of mycobacterial research in recent years. Concomitant advancements in genetics approaches for generating mutant strains and biochemical approaches in targeted enrichment have broadened proteomics applications. This suite of tools currently at our disposal has the capacity to answer questions at every step of information transfer in mycobacteria from genes to protein localization and behavior. Leveraged properly, classical approaches with proteomics gain critical insight into mycobacterial physiology and pathogenesis.
